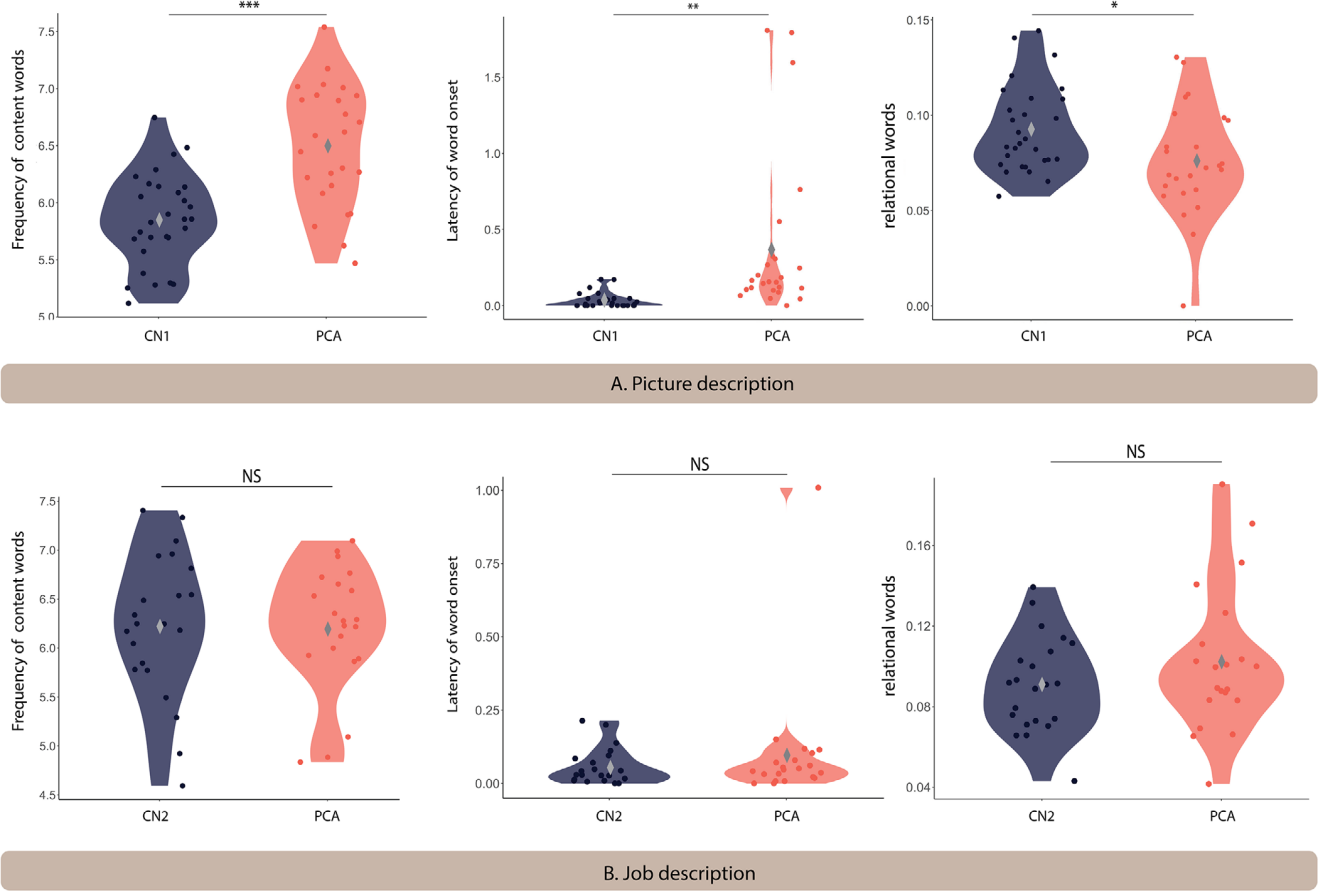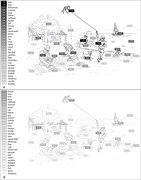# Into the Visual World of Patients with Posterior Cortical Atrophy through their Words: A Natural Language Processing Approach

**DOI:** 10.1002/alz.084032

**Published:** 2025-01-03

**Authors:** Neguine Rezaii, Daisy Hochberg, Megan Quimby, Bonnie Wong, Scott M McGinnis, Brad C Dickerson, Deepti Putcha

**Affiliations:** ^1^ Massachusetts General Hospital, Harvard Medical School, Boston, MA USA; ^2^ Frontotemporal Disorders Unit, Massachusetts General Hospital, Boston, MA USA; ^3^ Brigham and Women’s Hospital, Boston, MA USA; ^4^ Frontotemporal Disorders Unit, Department of Neurology, Massachusetts General Hospital and Harvard Medical School, Boston, MA USA

## Abstract

**Background:**

Posterior Cortical Atrophy (PCA) is a syndrome characterized by a progressive decline in higher‐order visuospatial processing, leading to symptoms such as space perception deficit, simultanagnosia, and object perception impairment. While PCA is primarily known for its impact on visuospatial abilities, recent studies have documented language abnormalities in PCA patients. This study aims to delineate the nature and origin of language impairments in PCA, hypothesizing that language deficits reflect the visuospatial processing impairments of the disease.

**Method:**

We compared the language samples of 25 patients with PCA with age‐matched cognitively normal (CN) individuals across two distinct tasks: a visually‐dependent picture description and a visually‐independent job description task. We extracted word frequency, word utterance latency, and spatial relational words for this comparison. We then conducted an in‐depth analysis of the language used in the picture description task to identify specific linguistic indicators that reflect the visuospatial processing deficits of PCA.

**Result:**

Patients with PCA showed significant language deficits in the visually‐dependent task, characterized by higher word frequency, prolonged utterance latency, and fewer spatial relational words, but not in the visually‐independent task. An in‐depth analysis of the picture description task further showed that PCA patients struggled to identify certain visual elements as well as the overall theme of the picture. A predictive model based on these language features distinguished PCA patients from CN individuals with high classification accuracy.

**Conclusion:**

The findings indicate that language is a sensitive behavioral construct to detect visuospatial processing abnormalities of PCA. These insights offer theoretical and clinical avenues for understanding and managing PCA, underscoring language as a crucial marker for the visuospatial deficits of this atypical variant of Alzheimer’s disease.